# Fructose-1, 6-diphosphate (FDP) as a novel antidote for yellow oleander-induced cardiac toxicity: A randomized controlled double blind study

**DOI:** 10.1186/1471-227X-10-15

**Published:** 2010-06-29

**Authors:** Indika Gawarammana, Fahim Mohamed, Steven J Bowe, Ashoka Rathnathilake, Shantha K Narangoda, Shifa Azher, Andrew H Dawson, Nick A Buckley

**Affiliations:** 1 South Asian Clinical Toxicology Research Collaboration, Department of Clinical Medicine, University of Peradeniya, Sri Lanka; 2 Centre for Clinical Epidemiology and Biostatistics, University of Newcastle, NSW, Australia; 3 Kurunegala Teaching Hospital, Kurunegala, North Western Province, Sri Lanka; 4 Medical Professorial Unit, POW Hospital Clinical School, University of NSW, Australia

## Abstract

**Background:**

Cardiac toxicity due to ingestion of oleander plant seeds in Sri Lanka and some other South Asian countries is very common. At present symptomatic oleander seed poisoning carries a mortality of 10% in Sri Lanka and treatment of yellow oleander poisoning is limited to gastric decontamination and atropine administration. The only proven effective antidote is digoxin antibodies but these are not available for routine use because of the high cost. The main objective of this study is to investigate the effectiveness of a new and inexpensive antidote for patients with life threatening arrhythmias due oleander poisoning.

**Method/design:**

We set up a randomised double blind clinical trial to assess the effectiveness of Fructose 1, 6 diphosphate (FDP) in acute yellow oleander poisoning patients admitted to the adult medical wards of a tertiary hospital in Sri Lanka. Patients will be initially resuscitated following the national guidelines and eligible patients will be randomised to receive either FDP or an equal amount of normal saline. The primary outcome measure for this study is the sustained reversion to sinus rhythm with a heart rate greater than 50/min within 2 hours of completion of FDP/placebo bolus. Secondary outcomes include death, reversal of hyperkalaemia on the 6, 12, 18 and 24 hour samples and maintenance of sinus rhythm on the holter monitor. Analysis will be on intention-to-treat.

**Discussion:**

This trial will provide information on the effectiveness of FDP in yellow oleander poisoning. If FDP is effective in cardiac glycoside toxicity, it would provide substantial benefit to the patients in rural Asia. The drug is inexpensive and thus could be made available at primary care hospitals if proven to be effective.

**Trial Registration:**

Current Controlled trial ISRCTN71018309

## Background

Cardiac glycoside toxicity is the most common type of plant poisoning in Sri Lanka and some other South Asian countries [1-3]. At present, symptomatic cardiac glycoside poisoning carries a mortality rate of 10% in Sri Lanka [1]. Cardiac glycosides inhibit the enzyme Na-K-ATPase of the cardiac myocyte and the conducting system and increase intracellular calcium concentrations. This rise in intracellular calcium may be a mechanism for ventricular arrhythmias [4]. These effects lead to increased automaticity and excitability both during early and late depolarization of the cardiac cell. Patients also develop very high serum potassium concentrations as a result of inhibition of Na-K-ATPase. Patients may develop arrhythmias and become hypotensive. Hypotension interferes with intracellular production of ATP through glycolysis, as lactate (produced due to anaerobic metabolism) inhibits the rate limiting enzyme phosphofructokinase. This in turn will further reduce the activity of Na-K-ATPase resulting in a vicious cycle.

FDP (CAS registry number 488-69-7; Merck monograph number 4297) is a phosphorylated sugar that is a normal physiological intermediary in glycolysis. It is produced from glucose by the action of phosphofructokinase during glycolysis and is in turn broken down into pyruvate. Phosphofructokinase activity is the main rate-limiting factor for ATP production from glucose under anaerobic conditions. Given intravenously, FDP is capable of being actively transported into cells and acting as an alternative energy source to glucose [5]. This can increase ATP production in circumstances where phosphofructokinase is inhibited (for example by lactate). The relative production of ATP is greater for FDP than glucose.

FDP has also been shown to stimulate Na-K-ATPase activity, and inhibit potassium efflux from myocardial cells [6,7]. It is hypothesised that these mechanisms may contribute to its activity in cardiac glycoside poisoning where Na-K-ATPase is inhibited and extracellular potassium is high. FDP also chelates ionised calcium. A decrease in ionised serum calcium and/or cardiac uptake of calcium may also be favourable,[8] given the high intracellular calcium that occurs in cardiac glycoside poisoning.

These theoretical benefits of FDP have been shown in an animal study done at the Mississippi School of Medicine, USA. This study showed evidence of effectiveness of FDP in dogs poisoned with a relative of the yellow oleander - the common or pink oleander. Cardiac arrhythmias in dogs (6/6;100%) treated with FDP (50mg/kg IV) reverted back to sinus rhythm within 30 minutes while the arrhythmias in the control animals killed one animal and did not revert over four hours in the other five at which time they were sacrificed [7]. There was also no rise in serum potassium in the group receiving FDP while marked hyperkalemia occurred in the control group [7].

We conducted a small phase II study of increasing IV bolus doses (30 to 250mg/kg) of FDP in patients with yellow oleander poisoning in Sri Lanka in 2006-7. The aim of that study was to find a safe dose of FDP that might be used to reverse cardio-toxicity. The agent was well tolerated with no evidence of any adverse effects attributable to FDP at any of the doses and an apparent reduction of around 0.5 mmol/L of potassium and 0.3mmol/L of calcium in the highest FDP dose group. All these doses were well within the range of doses used in previous human studies for other conditions [9].

### Previous Human Experience/safety profile for other indications

FDP has a well established safety profile in humans. It has been administered as a component of total parenteral nutrition (TPN) and has been used short-term in numerous experimental human studies. It has generally been shown to have favourable effects in these studies. For example, in individuals with coronary artery disease and heart failure, IV FDP increased cardiac work and reduced ventricular filling pressures [10].

The minimum dose where effects have been seen is around 25-50mg/kg [10]. Doses up to 250mg/kg IV have been used safely as a single dose [10-13] and 750 mg/kg IV as a cumulative dose (over 12 hours) [12]. The most pivotal study was a dose-ranging study of FDP to reduce ischemic injury post coronary artery bypass grafting. Five doses between 50mg/kg and 750 mg/kg in divided doses were tested with the optimal dose being 250mg/kg IV given over 30 minutes [12].

FDP is an approved product in Italy - the only safety concerns identified in the product information are a very rare risk of hypersensitivity reactions [14]. The approved dose range in Italy covers the doses to be used in this study.

## Methods/Design

### Design

The study is a double blind randomised controlled clinical trial with two parallel groups. The trials is designed to be compliant with the CONSORT Statement [15]

### Patients

**Figure 1:**
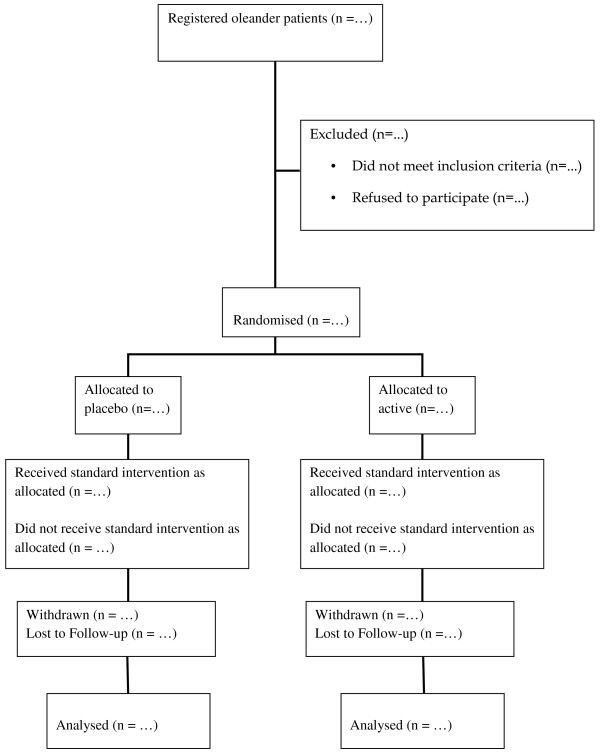
**Trial profile**.

All patients who present with a history of yellow oleander poisoning will be assessed to determine if they are eligible for the study. Those who meet the criteria will be approached to give their written informed consent, following which they will be randomised (Figure 1). Patients who do not initially meet the criteria will be reviewed regularly and approached if they meet the inclusion criteria at a later time point.

#### Inclusion criteria

patients who are admitted to adult medical wards, who are over the age of 16years, and who have any of the following manifestations of oleander induced cardiac toxicity:

• 2^nd ^degree heart block

• 3^rd ^Degree heart block

• Bradycardia with a heart rate of less than 40 beats/minute

• Any rhythm with a systolic blood pressure below 80 mm Hg

#### Exclusion criteria

Patients with documented ischemic or valvular heart disease, pregnant women and age under 16 years.

### Patient management

Standard oleander patient management guidelines will be followed. These are based on the national poisoning treatment guidelines[16]. The only difference between enrolled patients and those not enrolled will be the addition of FDP/placebo intervention.

### Trial intervention and study procedures

Patients randomised to the treatment arm will be treated with 250mg/kg loading dose of FDP (Esafosfina from Biomedical Foscama, Italy) over 20minutes followed by 6mg/kg/hr for 24 hours in addition to standard care. Patients randomised into the control arm will be treated with an equal volume (equal to the volume of FDP in the treatment arm) of 0.9% saline as a bolus and a 24 hour infusion. All attending doctors and nurses will be blinded to the treatment.

Clinical parameters such as systolic and diastolic blood pressure will be monitored for 24 hours. A Holter monitor will record the cardiac rhythm for 48 hours. All cardiac events will be recorded. If a serious cardiac rhythm abnormality recurs after an initial response to the bolus (within 2 hours of a bolus), a further bolus of 250mg/Kg of FDP (or equal volume of placebo) may be given at the discretion of the treating physician while the infusion will be maintained at the same rate.

### Randomisation

Randomisation is done using purpose designed computer software. The random sequence and allocation are concealed prior to randomisation. The program will randomise eligible patients in a 1:1 ratio. The allocation sequences are generated and encrypted independently by an IT consultant who has no role in patient recruitment, treatment and assessment.

The randomization will be performed by study pharmacists centrally. If a patient meets the inclusion criteria and gives consent, clinical research assistants will call the pharmacist with details such as name, hospital number and weight of the patients. Then the pharmacist will randomise the patients and prepare the placebo or active treatments for the study team. The allocation will only be known by the pharmacists who will have no other role in patient management and data collection. Intention-to-Treat analysis will be applied. That is, the analysis will include all randomised patients in the groups to which they were assigned, regardless of non-compliance, protocol deviations, withdrawal, and anything that happens thereafter [17]

### Outcomes

The primary outcome of this study is the reversion to sustained sinus rhythm with a heart rate >50 bpm within 2 hours of completion of bolus.

Secondary outcomes include:

1. Death

2. Change from baseline serum potassium on the 6, 12, 18 and 24 hour blood samples.

3. Time to revert and duration of reversion to sinus rhythm on the Holter monitor over the first 24 hours (reflecting the efficacy of bolus and infusion)

### Sample size

We are planning to recruit 240 symptomatic patients with yellow oleander poisoning admitted to Teaching Hospital, Kurunegala and National Hospital Sri Lanka, Colombo. In the study by Eddleston M et al [18], 2/32 (6.25%, 95% CI 1% - 19%) patients reverted to sinus rhythm spontaneously after 2 hours. Second degree and third degree heart block are markers of toxicity but may not be strong predictors of death (which may be due to myocardial toxicity rather than atrio-ventricular conduction block). Reversion to sinus rhythm is more likely with less severe second degree block. We would ideally have a sample size that ensured a significant number of third degree blocks which are less likely to revert spontaneously so that the effect of treatment in this sub-group can be seen. However, the proportions of the different grades of block are not well quantified and thus precise estimates of power in sub-groups are not possible.

We expect up to 15% spontaneous reversion by two hours and believe a 40% reversion in the active treatment arm would be clinically significant and likely to provide strong evidence that this would translate to a mortality benefit. This would require just 60 patients in each arm (α = 0.05, β = 0.8, missing data/dropout 10%). To increase the likelihood of recruiting enough patients with 3^rd ^degree heart block to see if the effect is observed in the most severely poisoned patients, and to account for possible differences between centres we intend to do a study of 120 patients in each arm.

### Study hypothesis and principal comparisons

The primary outcome of the study is to investigate if the addition of FDP (250mg/kg loading dose over 20 minutes followed by 6mg/kg/hr for 24 hours) to routine treatment will completely reverse serious arrhythmias within two hours. The study will also investigate the effect of FDP on the presence of abnormal rhythms and serum potassium and other electrolytes over 24 hours.

### Statistical Analysis

The Primary outcome (proportion with reversion to sinus rhythm) will be compared with the chi-squared test. Kaplan Meier curves will be constructed to demonstrate the cumulative reversion to sinus rhythm over time. A Cox proportional hazard model will be used to test the overall difference in reversion to sinus rhythm between treatments adjusted for baseline variables as necessary. A longitudinal statistical technique known as Generalised Estimating Equation (GEE) will be used to analyze changes in electrolytes (e.g. potassium) over time.

### Independent data monitoring and ethics committee (IDMEC)

The IDMEC will conduct the interim analysis and examine primary and secondary outcomes.

We intend to do a planned interim analysis once a total of 120 patients had been randomised (i.e. 60 in the treatment arm and 60 in placebo) which may lead to modification of or cessation of the trial as outlined below. The IDMEC will be asked specifically to comment on the need for subsequent modification of the trial protocol for the infusion. An infusion rate modification should be recommended if there is strong evidence of a response to the bolus which is not sustained by the infusion. An interim analysis at this stage will also be used to stop the trial if there is very strong evidence for efficacy. We have a 95% power to detect a 60% treatment effect over the expected placebo response of 15% without any loss of power for the overall study (α = 0.001, β = 0.95, missing data/dropouts 10%). If such a major response is noted the IDMEC will instruct the trial team to stop the trial.

### Ethics

This study is approved by the ethics research committee of University of Peradeniya and the Human Research Ethics Committee of the Australian National University. Written informed consent will be obtained from all patients in their native language (Sinhala or Tamil).

## Discussion

If FDP is proven to be effective, it will be a very useful treatment as this treatment is inexpensive and can be made readily available in rural hospitals of South Asia where poisoning with oleander seeds is very common[1-3]. There are no affordable alternative proven treatments for established arrhythmias for oleander poisoning.

Currently yellow oleander poisoning patients are managed with initial gastric decontamination methods such as gastric lavage, and activated charcoal, and administered atropine or occasionally isoprenaline to increase the heart rate. Anti-digoxin antibodies have proven to be effective [18] but are now prohibitively expensive for developing countries [19,20] and these are not available in Sri Lankan hospitals.

Clinical benefit of charcoal administration as a decontamination method has some conflicting results. Two methodologically different randomised control trials published so far have reported conflicting evidence of its benefit. De Silva et al in their double blind randomised control trial reported that multiple doses of activated charcoal (MDAC) 50 g 6 hourly for 72 hours reduced mortality and occurrence of life-threatening arrhythmias[21]. In contrast Eddleston et al reported no reduction in mortality in the subgroup of oleander patients (n = 1647) who were treated with either single dose of activated charcoal (SDAC) or MDAC or no activated charcoal[22]. It would be very difficult to draw a definite conclusion on the efficacy of MDAC based on substantial difference between these two RCTs [19].

Atropine is the most widely used agent in treating oleander induced bradyarrhythmias [1]. However there is no evidence of any benefit of atropine in such conditions [23]. Patients with slow heart rate (below 40 beats/minutes) are also routinely transferred to tertiary hospital where the facilities for transvenous cardiac pacing are available. However there has been no clinical trial to evaluate the effectiveness of this intervention and many patients die despite pacing [19].

## Competing interests

The authors declare that they have no competing interests.

## Authors' contributions

IG, NAB and AHD designed the protocol. IG is in charge of implementation of the study. FM is in charge of randomization, logistics, auditing and collating data. SJB advised on the statistical analysis plan. AR, SKN, and SA are in charge of the clinical management of patients.

## Pre-publication history

The pre-publication history for this paper can be accessed here:

http://www.biomedcentral.com/1471-227X/10/15/prepub
